# Pirfenidone for Idiopathic Pulmonary Fibrosis: A Systematic Review and Meta-Analysis

**DOI:** 10.1371/journal.pone.0136160

**Published:** 2015-08-26

**Authors:** Carlos Aravena, Gonzalo Labarca, Carmen Venegas, Alex Arenas, Gabriel Rada

**Affiliations:** 1 Department of Respiratory diseases, Faculty of Medicine, Pontificia Universidad Católica de Chile, Santiago, Chile; 2 Department of Internal Medicine, Faculty of Medicine, Pontificia Universidad Católica de Chile, Santiago, Chile; 3 Evidence Based Health Care Program, Faculty of Medicine, Pontificia Universidad Católica de Chile, Santiago, Chile; 4 Epistemonikos foundation, Santiago, Chile; Imperial College, London, UNITED KINGDOM

## Abstract

Idiopathic pulmonary fibrosis (IPF) is a progressive disease with poor prognosis. In the last decades pirfenidone an anti-inflammatory and anti-fibrotic agent has shown benefit in inhibit collagen production and has also demonstrated benefit in decline progression in IPF in physiological outcomes as Forced vital capacity (FVC), in clinical outcomes such as progression free survival (PFS) and a benefit in mortality but no in clinically relevant outcomes as exacerbations or worsening of IPF. Methods: We conducted a systematic review to evaluate the effectiveness of physiological and clinical outcomes of pirfenidone compared to placebo in IPF. We performed a search with no language restriction. Two researchers performed literature search, quality assessment, data extraction and analysis. And was performed a summary of findings table following the GRADE approach. Results: We included 5 RCTs (Randomized controlled trials) in analysis. The meta-analysis resulted in a decrease in all cause-mortality (RR 0.52 IC 0.32–0.88) and IPF related mortality (RR 0.32 IC 0.14–0.75); other outcomes evaluated were worsening of IPF (RR 0.64 IC 0.50–0.83) and acute exacerbation (RR: 0.72 IC 0.30–1.66 respectively). Also there was a decrease in progression free survival (PFS) (RR 0.83 IC 0.74–0.92) compared to placebo. Conclusions: We observed significant differences in physiologic and clinically relevant outcomes such as reduction in all-cause mortality, IPF related mortality, worsening and exacerbation of IPF and PFS. So pirfenidone treatment should be considered not only for its benefits in pulmonary function tests but also by its clinically relevant outcomes.

## Introduction

Idiopathic pulmonary fibrosis (IPF) is a rare and progressive disease of unknown etiology characterized by dyspnea and deterioration of lung function, with poor quality of life and a median survival time of about 3 years[1]. Several drugs have failed to demonstrate utility in this disease[2], or have been associated with significant adverse effects or even higher mortality[3].

Pirfenidone (5-methyl-1-phenyl-2- [1H]-pyridone) is an agent that combines anti-inflammatory and antifibrotic effects, acting through the regulation of TNFα and TNFβ’s pathways, as well through modulation of cellular oxidation[4,5]. Since the late nineties, some studies have showed that pirfenidone inhibits fibroblast proliferation and collagen synthesis and deposition, both in vitro and in animal models [6–8]. Initial open-label trials showed therapeutic potential for IPF [9,10], and the first prospective clinical studies demonstrated slowing the deterioration of lung function measured as change in lowest SPO2 during 6-minute exercise test (6MET) and vital capacity (VC) (11,12). Based on these findings, Japan approved in 2008 the use of pirfenidone to treat mild to moderate IPF.

The CAPACITY programme (PIPF 004 and 006) included patients with mild to moderate idiopathic pulmonary fibrosis, which was treated for 72 weeks. The PIPF 004 trial, which compared pirfenidone vs. placebo, showed reduction in FVC decline. The PIPF 006 trial, however, did not show significant differences between groups treated with pirfenidone after 48 weeks[11,12]. Based in the pooled analysis of both studies the European Union approved the use of this drug in 2011. Then a Cochrane meta-analysis of these studies besides an effect in FVC, it also found a significant 30% increase in progression free survival, although no effect on mortality was found and other clinically relevant outcomes were not evaluated[13]. A network meta-analysis published in 2014 also showed a possible significant effect on FVC but clinically relevant outcomes were not analyzed.[14]

FDA requested additional evidence to support the use of pirfenidone in USA. Intended to assess and confirm the efficacy and safety of pirfenidone the ASCEND study[15] was performed in IPF patients who received pirfenidone 2403 mg/day vs. placebo for 52 weeks, they observed a relative reduction of 47.9% in the proportion of patients who had an absolute decline of ≥10% in the percentage of the predicted FVC or who died. Pirfenidone also increased the proportion of patients with no decline in FVC and reduced the decline in 6MWT. In addition the ASCEND study pooled all mortality data, incorporating PIPF 004 and 006 studies in the analysis resulting in a significant decrease in this outcome.

After the publication of this last trial pirfenidone was approved by FDA for the treatment of IPF but despite this study showed a mortality benefit at this time the Division of Drug Information in the FDA's Center for Drug Evaluation and Research only consider physiological outcomes (change in FVC) as the efficacy endpoint that was met but no other clinically meaningful outcomes. [16]

The aim of this study is to assess the efficacy and security of pirfenidone on several clinical (including mortality, acute exacerbations and worsening of IPF) and physiological outcomes in IPF, conducting a systematic review and meta-analysis of the current published literature.

## Material and Methods

### Literature search and clinical eligibility criteria

Two reviewers independently searched the following electronic databases: PubMed, Lilacs, Clinical Trials Registry Platform (ICTRP), clinicaltrials.gov and the Cochrane Controlled Trials Register up to October 30th of 2014. We also searched in two specialized evidence databases: TRIP database and Epistemonikos[17], which conduct searches for systematic reviews in several other databases following PRISMA statement[18].

For maximum sensitivity, we used Mesh term: “pirfenidone” alone and in combination with others term such as: ("pirfenidone" [Supplementary Concept] OR "Anti-Inflammatory Agents, Non-Steroidal"[Mesh]) AND ("Pulmonary Fibrosis"[Mesh]). All meeting abstracts of European respiratory society (ERS) from 2010 to 2014, American thoracic society (ATS) and American college of chest physicians (ACCP) from 2010 to 2014 were searched.

Studies were eligible for inclusion if they met the following criteria: The patients in the selected studies were older than 18 years, and had idiopathic pulmonary fibrosis; the studies were RCTs and compared Pirfenidone against placebo, and had at least one or more clinical outcomes.

Full articles were retrieved, when titles and/or abstracts met this objective. A manual cross-reference search of relevant articles was conducted.

There was no language restriction on publications. Discordance about study inclusion between the two reviewers (CA and GL) was resolved through discussion until 100% agreement was reached on the final interpretation of the data.

### Quality assessment of retrieved articles

The selected studies were appraised by two reviewers (AA and GL), who independently assessed their quality using the methods recommended in the Cochrane Handbook for Systematic Reviews of Interventions.[19]

### Outcome measure

The included outcomes in the analysis were: 1) Change in all cause- mortality 2) Change in IPF related mortality 3) Progression-free Survival (PFS) 4) Decrease in predicted Forced Vital Capacity (FVC) 5) Worsening of Idiopathic pulmonary fibrosis 6) Acute exacerbation 7) Change in Six-Minute Walk Test (6MWT) Distance 8) Adverse Effect (all included adverse events, skin related adverse events and change in aminotransferases. In addition, a subgroup analysis evaluating worsening of IPF (Japanese and no-Japanese studies) was performed.

### Data extraction and analysis

Data extraction and analysis was performed by two independent reviewers (GL and AA). The studies were tabulated and methodologically evaluated to assess homogeneity. Any heterogeneity between the studies would not be justified to pool the assessed outcomes. A customized data-extraction form, as described in the Cochrane Handbook for Systematic Reviews of Interventions was used to record the duration of the trial, sample size, dropouts, and effect of interventions.

Quantitative data were analyzed using the Cochrane Review Manager (RevMan) version 5.2 software. Summary estimates, including 95% CIs, were calculated. For continuous outcome data, means and standard deviations were used to calculate a weighted mean difference (WMD). For dichotomous outcomes, the RR was calculated.

Statistical heterogeneity was tested using the Q statistic of the c2 value test and I2 test. Fixed-effects models were used, unless significant evidence of statistical heterogeneity or clinical diversity was found. For results showing significant heterogeneity (I2>50%)[19], A random-effects meta-analysis was performed by the Der Simoniane Laird method. Outcome measures were assessed on an intent-to-treat basis. A p value of <0.05 was considered statistically significant. A sensitivity analysis was performed and Publication bias was assessed by visually inspecting a funnel plot.

Finally, we created a summary of findings table following the GRADE approach, using GRADEpro software.

## Results

### Summary of main results

**Fig 1:**
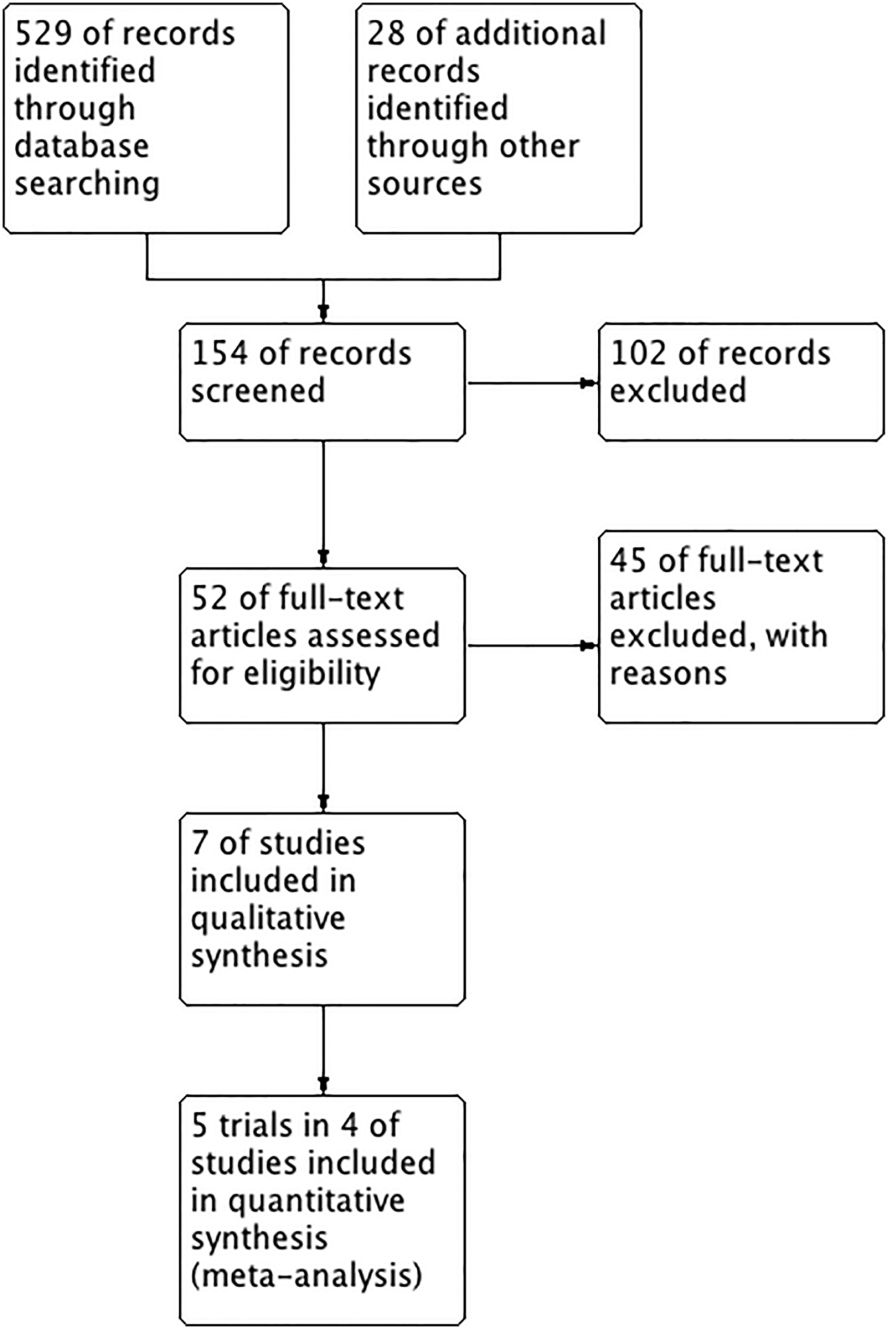
PRISMA flow diagram.

**Fig 2:**
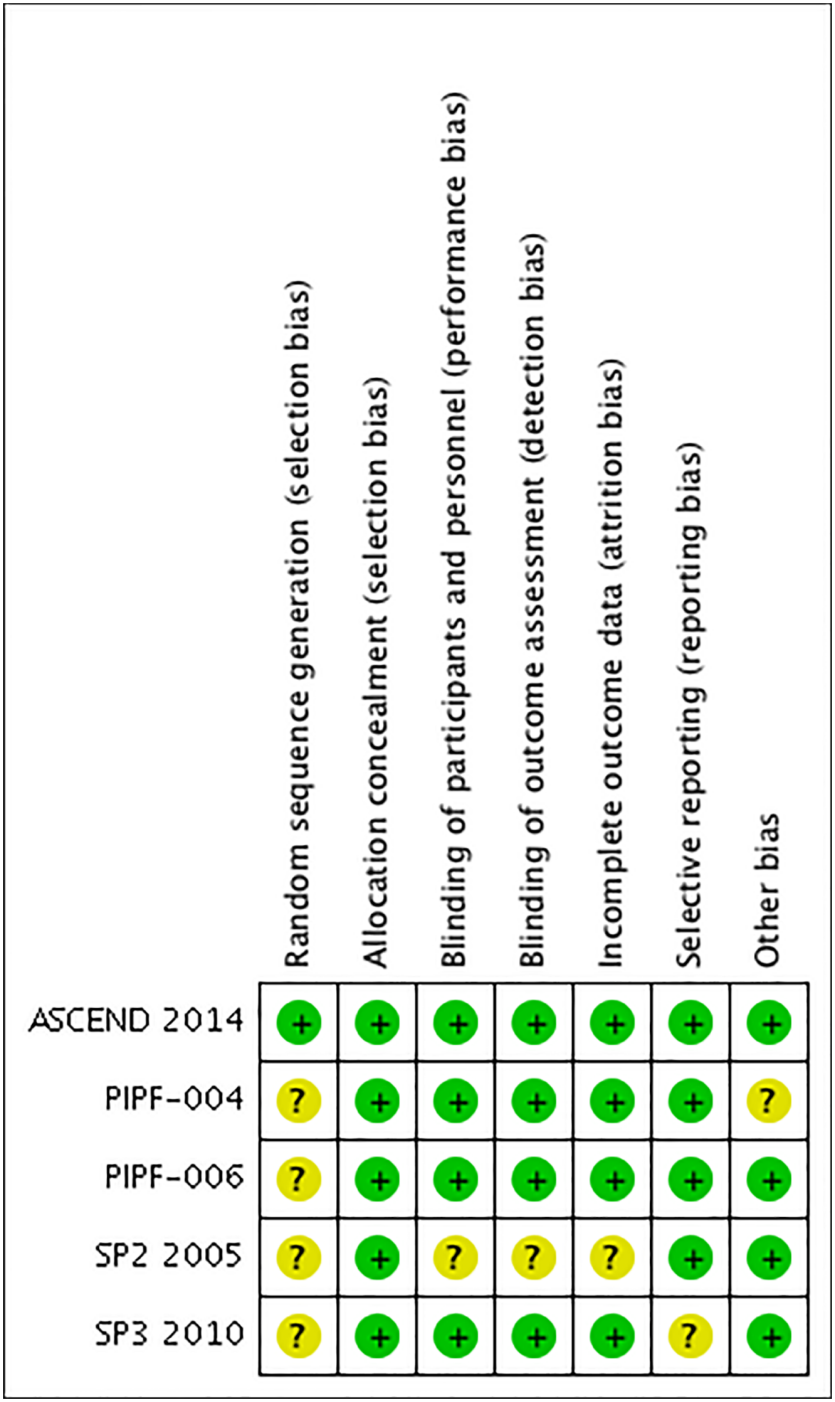
Risk of bias summary: review authors' judgment’s about each risk of bias item for each included study.

A total of 529 studies were identifiable for library search and 28 were identifiable for others sources. A total of 5 RCTs included in four publications were analyzed. See Table 1 and Fig 1 for a summary of the characteristics of these studies. Excluded studies are shown in supplemental data (S1 Table)[9,20,21]. Quality assessment and risk of BIAS are shown in Fig 2.

**Table 1:** Characteristics of included studies in this systematic review.

Trial	Year	N° subjects (intervention/placebo)	Type of studies	Intervention	Comparison	Primary outcome	GRADE
CAPACITY (PIPF 004)	2011	174/174	Parallel	Pirfenidone 1197 mg/day or pirfenidone 2403 mg/day	Placebo pills	Change from baseline to week 72 in predicted FVC	MODERATE
CAPACITY (PIPF 006)	2011	171/173	Parallel	Pirfenidone 2403 mg/day	Placebo pills	Change from baseline to week 72 in predicted FVC	MODERATE
SP2	2005	72/35	Parallel	Pirfenidone 200 mg TID for 2 days, 400 mg TID for 2 days and 600 mg TID for 3 days	Placebo pills	Change in the lowest spo2 during 6 mwt	LOW
SP3	2010	163/104	Parallel	Pirfenidone in stepwise doses; 1800 mg/day in high dose and 1200 mg/day in low dose	Placebo pills	Change from baseline to week 52 in predicted FVC	LOW
ASCEND	2014	278/277	Parallel	Pirfenidone 2403 mg/day	Placebo pills	Change from baseline to week 52 in predicted FVC	MODERATE

### Effect of interventions

The summary of findings table (GRADE) is shown in Table 2. Funnel plots from all comparisons included in our meta-analysis are shown as supplemental data S1–S10 Figs and PRISMA checklist is shown on S1 PRISMA Checklist.

**Table 2:** Summary of finding form Pirfenidone for idiopathic pulmonary fibrosis.

Outcomes	Anticipate absolute effects (Study population) (95% CI)		Relative Effect	NO of participants	Quality of the evidence (GRADE)
	Risk with placebo	Risk with Pirfenidone			
All cause-mortality	67 per 1000	36 per 1000 (22 to 59)	RR 0.53 (0.32 to 0.88)	1247 (3 RCTs)	⨁⨁⨁◯ MODERATE1
Progression free-survival	442 per 1000	372 per 1000 (332 to 416)	RR 0.83 (0.75 to 0.94)	728 (3 RCTs)	⨁⨁⨁◯ MODERATE1
Acute exacerbation	26 per 1000	15 per 1000 (5 to 47)	RR 0.59 (0.19 to 1.84)	235 (2 RCTs)	⨁⨁◯◯ LOW1,2
Worsening of IPF	168 per 1000	107 per 1000 (84 to 139)	RR 0.64 (0.50 to 0.83)	1615 (5 RCTs)	⨁⨁⨁◯ MODERATE1
Change on 6MWT	417 per 1000	308 per 1000 (267 to 358)	RR 0.74 (0.64 to 0.86)	1236 (3 RCTs)	⨁⨁⨁⨁ HIGH
Change on aminotransferases	30 per 1000	68 per 1000 (40 to 115)	RR 2.26 (1.33 to 3.83)	764 (5 RCTs)	⨁⨁⨁◯ MODERATE1

1: Non primary outcome from RCTs, 2: High heterogeneity; 6MWT: Six minutes walk test; RCT: Randomized controlled trial; RR: Risk ratio; CI: confidence interval.

### Mortality

**Fig 3:**
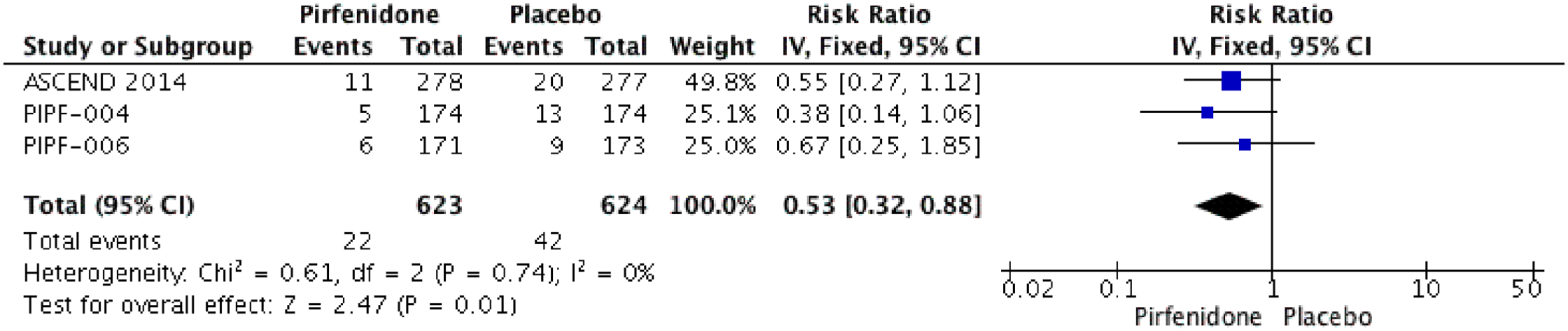
Comparison 1. All cause-mortality at week 52.

**Fig 4:**
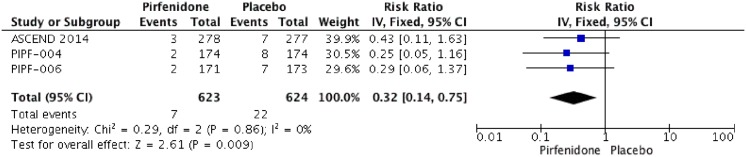
Comparison 2. Idiopathic pulmonary fibrosis (IPF) related mortality at week 52.

Three RCTs (1247 patients) were identified that reported the effect of pirfenidone and mortality (ASCEND 2014; PIPF004 2011 and PIPF006 2011)[11,15,22,23]. The meta-analysis includes 623 patients in intervention group and 624 in placebo group (Figs 3 and 4). Pirfenidone compared to placebo decreased all cause-mortality (RR: 0.53 IC 0.32–0.88, I2:0%) and IPF related mortality (RR: 0.32, IC 0.14–0.75; I2: 0%) at week 52. We rated the quality of evidence as moderate, because this outcome was not of primary interest in the different studies (indirectness).

### Progression-free Survival (PFS)

**Fig 5:**
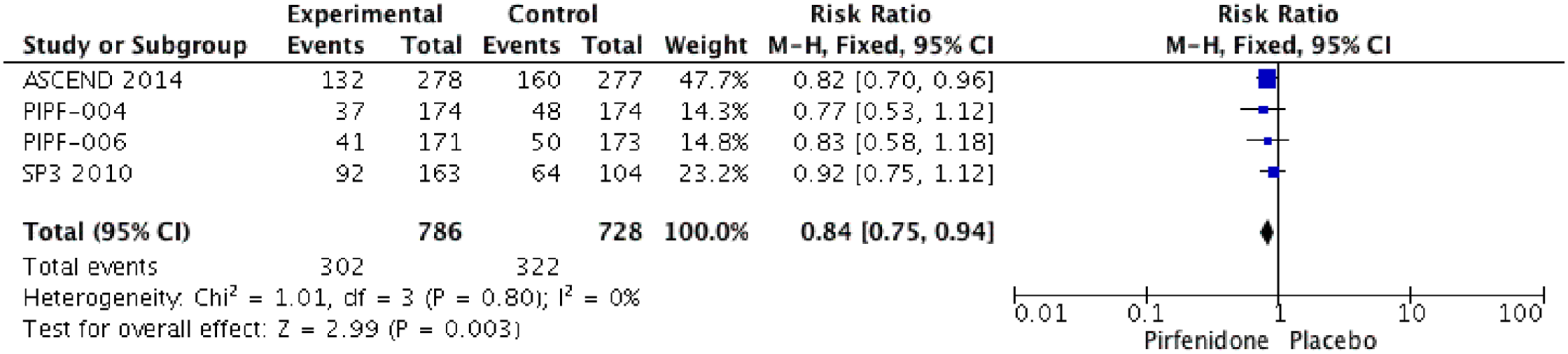
Comparison 3. Change on Progression-free Survival (PFS).

Five RCTs (PIPF004, PIPF006, ASCEND, SP3 and SP2) were identified that reported the effect of pirfenidone and Progression-free Survival. Pooled data from all studies were evaluated at week 52. When PFS was not reported at week 52, data were extracted from Kaplan-Mayer curves and number of events at this week. The meta-analysis includes 850 patients in intervention group and 863 in placebo group (Fig 5). Pirfenidone decreased PFS at week 52 (RR: 0.83 IC 0.74–0.92, I2:0%) compared to placebo. We rated the quality of evidence as moderate, because of indirectness.

### Acute exacerbation of IPF

**Fig 6:**
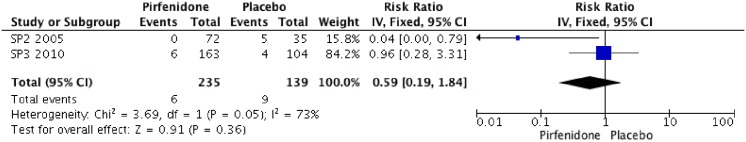
Comparison 4. Acute exacerbation.

Four RCTs (SP2, SP3, PIPF004, PIPF006) reported acute exacerbation of IPF. However, in CAPACITY trial (PIPF005 and PIPF 006) exacerbation was reported as “time to acute exacerbation” was part of a composite secondary endpoint and the events were not analyzed separately. The meta-analysis includes 235 patients in intervention group and 139 in placebo group (Fig 6). Pirfenidone do not improves acute exacerbation of IPF with a RR of 0.59 (IC 0.19–1.84, I2: 73%) compared to placebo. We rated the quality of evidence as LOW, because of indirectness and imprecision between results.

### Worsening of IPF

**Fig 7:**
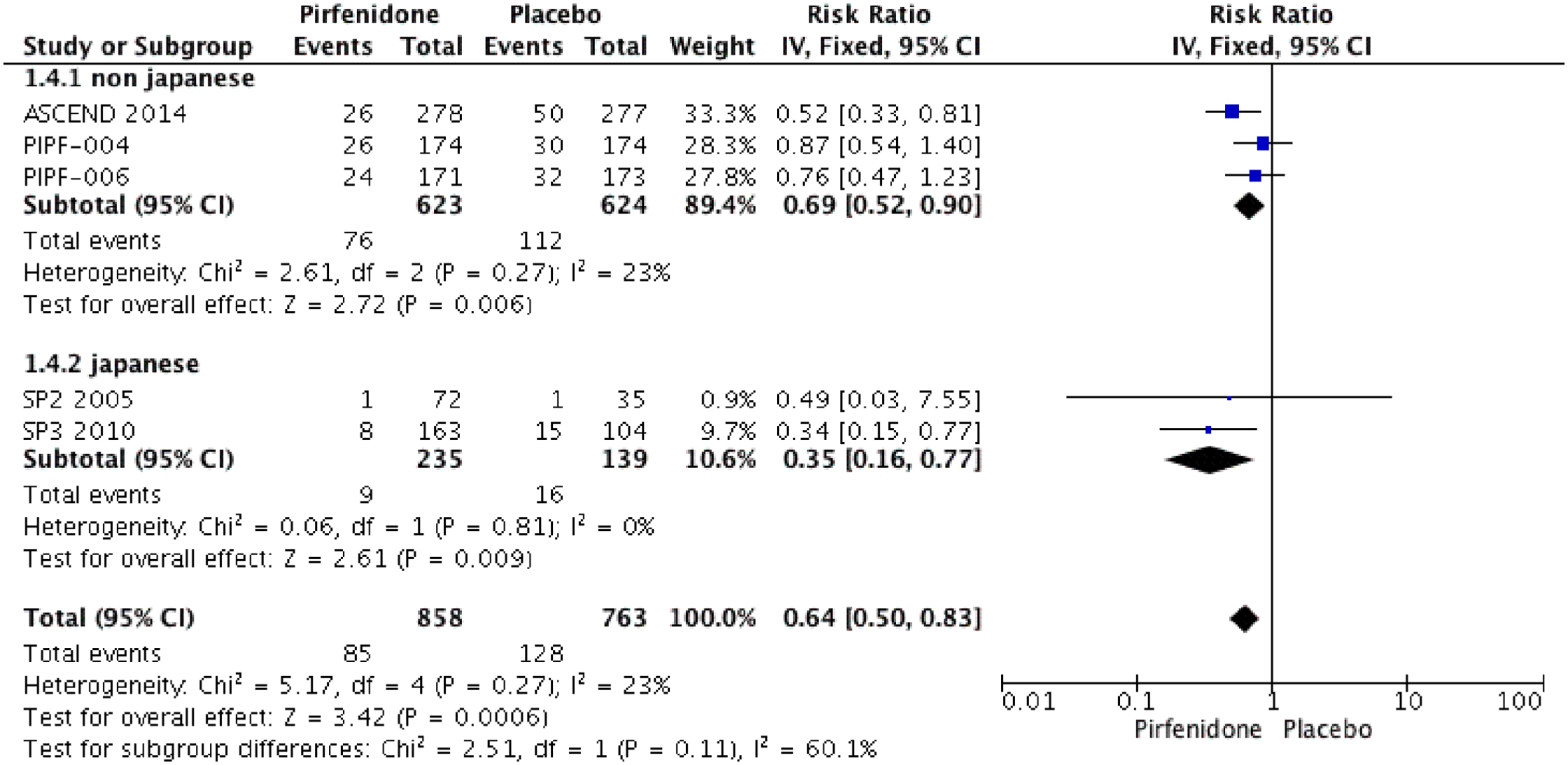
Comparison 5. Worsening of IPF, subgroup analysis from Japanese and non- Japanese studies.

Three RCTs (PIPF004, PIPF006, ASCEND) reported worsening of IPF. This secondary endpoint evaluated in 3 RCTs is related to a composite outcome that included acute IPF exacerbation, IPF related death, lung transplantation or respiratory hospitalization. The meta-analysis includes 786 patients in intervention group and 728 in placebo group (Fig 7). Pirfenidone improves worsening of IPF with a RR of 0.84 (IC 0.74–0.85, I2:0%). compared to placebo. We rated the quality of evidence as moderate, because of indirectness.

### Predicted Forced Vital Capacity (FVC)

**Fig 8:**
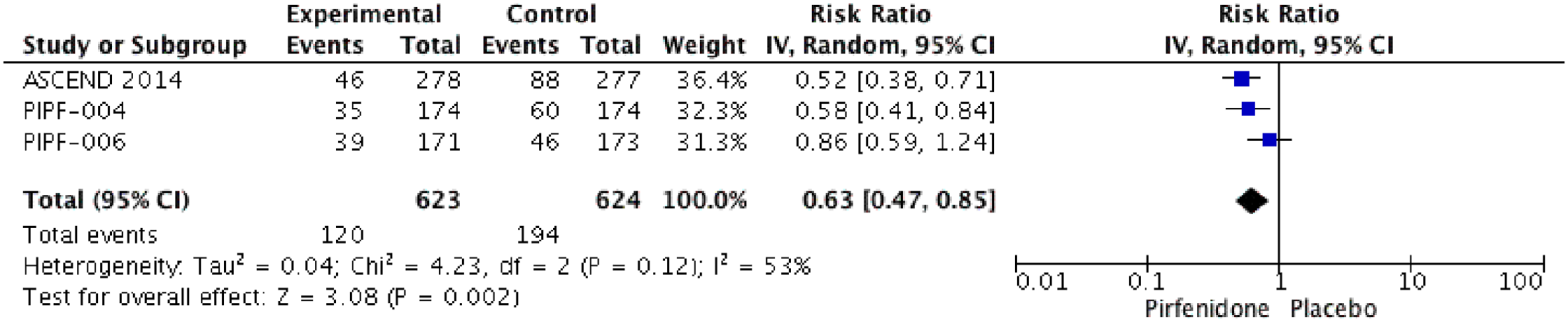
Comparison 6. Change >10% of Forced Vital capacity (FVC).

Five RCTs (PIPF004, PIPF006, ASCEND, SP3 and SP2) were identified that reported the effect of pirfenidone and FVC or vital capacity (VC). In three RCTs (ASCEND, SP3 and SP2) change of percentage of predicted forced vital capacity >10% were reported. The meta-analysis includes 623 patients in intervention group and 624 in placebo group (Fig 8). Pirfenidone decrease the risk of change >10% of FVC with a Risk ratio of 0.63 (IC 0.47–0.85%, I2: 53%) compared to placebo. We rated the quality of evidence as Moderate due imprecision.

### Change in Six-Minute Walk Test (6MWT) Distance

**Fig 9:**
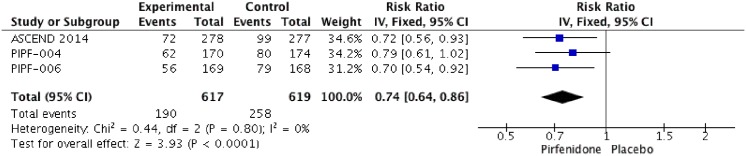
Comparison 7. Change on 6-minutes walked test (6MWT) distance.

Three RCTs (PIPF004, PIPF006, ASCEND) reported change in 6MWT distance. The meta-analysis includes 617 patients in intervention group and 619 in placebo group (Fig 9). Pirfenidone improves 6MWT distance with a RR of 0.74 (IC 0.64–0.86, I2:0%) compared to placebo. We rated the quality of evidence as high.

### Adverse events

**Fig 10:**
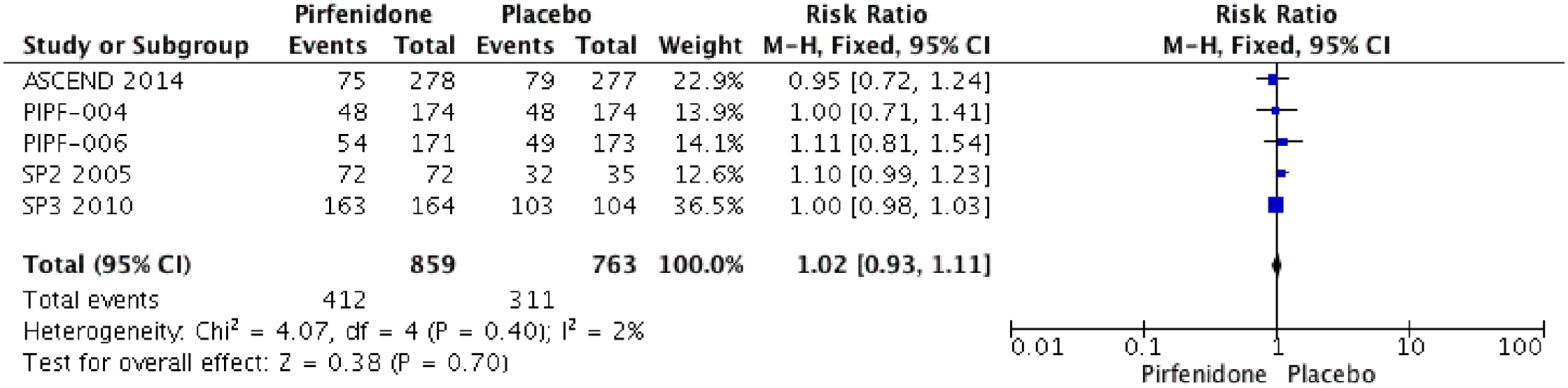
Comparison 8. Adverse events.

**Fig 11:**
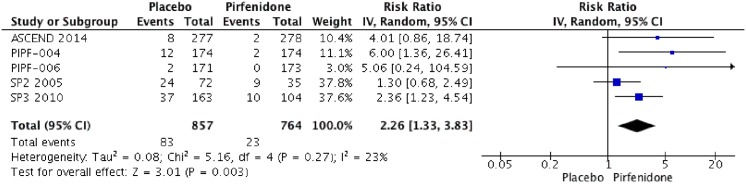
Comparison 9. Change on aminotransferase secondary to treatment.

**Fig 12:**
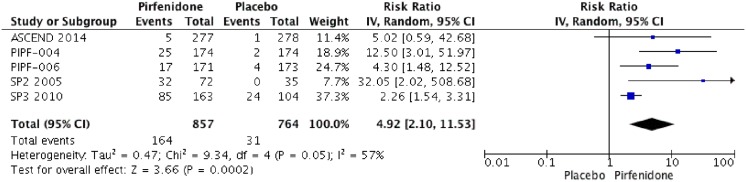
Comparison 10. Skin related adverse events.

Five RCTs (PIPF004, PIPF006, ASCEND, SP3 and SP2) were identified that reported the effect of pirfenidone and adverse events. Pooled data from all studies were evaluated at the end of each trial. The meta-analysis includes 857 patients in intervention group and 766 in placebo group (Fig 10). Pirfenidone is not associated with severe adverse events RR: 1.02 (IC 0.93–1.11, I2: 2%) compared to placebo. But other adverse events such as photosensitivity (RR: 4.92; IC 2.10–11.53, I2: 57%) or change on aminotransferases (RR: 2.26; IC 1.33–3.83, I2: 23%) were more frequent than placebo. We rated the quality of evidence as Moderate, because of imprecision between results. Characteristics of treatment-emergent adverse events are shown in Figs 11, 12 and Table 3.

**Table 3:** Summary of adverse events.

	SP2	SP3	(PIPF-004 and PIPF-006)	ASCEND
Nausea	72/2	ND	125–60	100/37
Rash	ND	ND	111/40	78/24
Dyspepsia	ND	ND	66/26	41/49
Vomiting	ND	ND	47/15	36/24
Photosensitivity	32/0	85/24	37/6	5/1
Anorexia	23/2	24/3	37/13	44/18
Decreased appetite	22/3	7–0	30/10	49/17
Weight loss	ND	ND	28/12	35/22
Asthenia	ND	ND	24–13	58/48
Upper airway infection	12/3	04/9	ND	61/56
Elevation in aminotransferase	24/9	37/10	14/2	8/2

Right: Pirfenidone group; left: placebo group N.D: No data.

## Discussion

Idiopathic pulmonary fibrosis is a chronic, progressive disorder that affects predominantly older patients and has poor prognosis with death rates even worse than many cancers[24].

Before the publication of ASCEND study there was no clear consensus about treatment for this disease. In the 2011 ATS/ERS Guidelines[25], despite have some of data from CAPACITY programme and the potential benefits in physiological outcomes the authors did not recommend the use of any specific pharmacologic therapy for patients with IPF, and only suggest support and adjunctive management, like long-term oxygen, pulmonary rehabilitation, gastroesophageal reflux control and palliative care. While this may be caused because the guidelines are out of date, it may also be due to lack of clinically significant outcomes to strongly support the use of a drug. Related to this reflection the use of systematic reviews with meta-analysis looking for clinical meaningful outcomes may be helpful to develop new clinical practice guidelines.

Unfortunately, previously used drugs (prednisone, azathioprine and N-acetyl cysteine among others) are nowadays discredited after the publication of trials that showed higher mortality rates and/or no improvement in slowing decline of lung function[3,26,27].

Pirfenidone has been a promising drug after initial research that showed its anti-inflammatory, anti-fibrotic and anti-oxidant activity[28–30]. After those in vitro and animal trials, Phase 2 studies and Phase 3 trials were conducted to further elucidate the impact of pirfenidone in patients with IPF[11,23]. The first pooled analysis of these data showed modest results, mostly in lung function, with no benefits in clinically relevant outcomes as mortality, although improvements in progression-free survival and mortality appear promising. This inconsistent effect may be due the limited number of patients, disease phase (recent or former diagnosis) or insufficient follow up. After the recent publication of ASCEND study that confirmed the significance in different physiological and clinical outcomes, this provided more power to determine the potential benefit in mortality that affects these patients.[29]

In this meta-analysis, we observed benefits in several outcomes. The slower decline in lung function test can be seen in the change in FVC and in the change in the 6-minute walk distance the first two of them have a high quality grade, so the estimate of effect is reliable. We also observed differences in clinically relevant outcomes such as reduction in all-cause mortality, IPF related mortality, worsening of IPF and progression free survival; but no benefit on acute exacerbation of IPF: these outcomes have a moderate quality grade and we should consider that future studies may change the estimate of effect. The main limitations of this Systematic review were: the scarce studies that evaluate this intervention; the trials analyzed in this study were performed in different populations; and there were different inclusion criteria in all of them. For example some differences between ASCEND and CAPACITY studies were: ASCEND patients had a poorer baseline lung function, were less likely to have concurrent airflow limitation, had a more likely refined population because underwent to a central review with radiological and pathological analysis prior to enrolment and patients in ASCEND were treated for 52 as opposed to 72 weeks[31]. In the other hand in statistical analysis we did not find significant heterogeneity in several outcomes included all clinical outcomes.

In relation to adverse effects treatment with pirfenidone has been well tolerated in all phase 3 trials. The main adverse effects are photosensitivity (12% in CAPACITY Trials and 51% in the Japanese study), gastrointestinal symptoms and dizziness; the last two were reported only in the CAPACITY program. In this meta-analysis, there is no difference in relative risk of severe adverse effects, but we must consider that is a low quality estimator, so Phase IV clinical trials should be conducted to answer this specific question.

The included systematic review was prepared according to the guidelines of The Cochrane Collaboration and was of high quality in most respects. The quality of the evidence reported by the primary studies in the included reviews was rated using the Cochrane methods and the body of evidence was rated using GRADE and ranged from very low to moderate. The main reasons for the quality of the evidence being downgraded were bias in the primary studies (inadequate reporting of allocation concealment and randomization methods, lack of blinding) and imprecision. The evidence was frequently restricted to a single small trial. No biases were identified during the overview process and no other systematic reviews were identified. We consider this list as the actual body of evidence for this question.

## Conclusion

Finally, given the results of this Systematic Review on a poor prognosis disease without previously proven treatment, the associated lower risk and benefits in physiological and clinically relevant outcomes, and considering a future RCT with a mortality primary endpoint is not feasible because the necessary population size, duration and cost. [32] The use of this drug should be highly considered. However, according to our data, this drug does not decrease the risk of acute exacerbation, but more evidence from future RCT is need to improve this outcome.

## Supporting Information

S1 Fig Funnel plot from all cause- mortality. (TIF)

S2 Fig Funnel plot from IPF related dead. (TIF)

S3 Fig Funnel plot from progression free survival. (TIF)

S4 Fig Funnel plot from acute exacerbation. (TIF)

S5 Fig Funnel plot from worsening of IPF. (TIF)

S6 Fig Funnel plot from change in FVC. (TIF)

S7 Fig Funnel plot from 6MWT. (TIF)

S8 Fig Funnel plot from all adverse events. (TIF)

S9 Fig Funnel plot from change on aminotransferases. (TIF)

S10 Fig Funnel plot from skin related adverse events. (TIF)

S1 PRISMA Checklist PRISMA checklist. (DOCX)

S1 Table Excluded studies of this systematic review. (DOCX)
