# Correction: Pirfenidone for Idiopathic Pulmonary Fibrosis: A Systematic Review and Meta-Analysis

**DOI:** 10.1371/journal.pone.0140288

**Published:** 2015-10-06

**Authors:** Carlos Aravena, Gonzalo Labarca, Carmen Venegas, Alex Arenas, Gabriel Rada

There are errors in the ninth and tenth sentences of the Abstract. These sentences should read: Also there was a decrease in the risk of progression (RR of PFS: 0.82 IC 0.73–0.92) compared to placebo. Conclusions: We observed significant differences in physiologic and clinically relevant outcomes such as reduction in all-cause mortality, IPF related mortality, worsening of IPF and improvement of PFS. So pirfenidone treatment should be considered not only for its benefits in pulmonary function tests but also by its clinically relevant outcomes.

There are multiple errors in the Results described below.

The third and fourth sentences of the “Progression-free Survival (PFS)” section should read: The meta-analysis includes 786 patients in intervention group and 728 in placebo group (Fig 5). Pirfenidone decreased the risk of progression (RR of PFS: 0.82 IC 0.73–0.92, I2:22%) compared to placebo. We rated the quality of evidence as moderate, because of indirectness.

The third sentence of the “Worsening of IPF” section should read: The meta-analysis includes 858 patients in intervention group and 763 in placebo group (Fig 7). Pirfenidone improves worsening of IPF with a RR of 0.64 (IC 0.50–0.83, I2:23%) compared to placebo.

The second sentence of the “Adverse events” section should read: The meta-analysis includes 859 patients in intervention group and 763 in placebo group (Fig 10).

There is an error in the third sentence of the fourth paragraph of the Discussion. It should read: We also observed differences in clinically relevant outcomes such as reduction in all-cause mortality, IPF related mortality, worsening of IPF and risk of progression; but no benefit on acute exacerbation of IPF.

There are errors in the fourth and fifth columns of Table 2. Please see the corrected Table 2 here.

**Table 2:** Summary of finding form Pirfenidone for idiopathic pulmonary fibrosis. 1: Non primary outcome from RCTs, 2: High heterogeneity; 6MWT: Six minutes walk test; RCT: Randomized controlled trial; RR: Risk ratio; CI: confidence interval

Outcomes	Anticipate absolute effects (Study population) (95% CI)		Relative Effect	NO of participants	Quality of the evidence (GRADE)
	Risk with placebo	Risk with Pirfenidone			
All cause-mortality	67 per 1000	36 per 1000 (22 to 59)	RR 0.53 (0.32 to 0.88)	1247 (3 RCTs)	⨁⨁⨁◯MODERATE1
Progression free-survival	442 per 1000	372 per 1000 (332 to 416)	RR 0.82 (0.73 to 0.92)	1514 (4 RCTs)	⨁⨁⨁◯MODERATE1
Acute exacerbation	26 per 1000	15 per 1000 (5 to 47)	RR 0.59 (0.19 to 1.84)	374 (2 RCTs)	⨁⨁◯◯LOW1,2
Worsening of IPF	168 per 1000	107 per 1000 (84 to 139)	RR 0.64 (0.50 to 0.83)	1621 (5 RCTs)	⨁⨁⨁◯MODERATE1
Change on 6MWT	417 per 1000	308 per 1000 (267 to 358)	RR 0.74 (0.64 to 0.86)	1236 (3 RCTs)	⨁⨁⨁⨁HIGH
Change on aminotransferases	30 per 1000	68 per 1000 (40 to 115)	RR 2.26 (1.33 to 3.83)	1621 (5 RCTs)	⨁⨁⨁◯MODERATE1

Fig 5 and its caption are incorrect. Please view Fig 5 and see its complete, correct caption here.

**Fig 5:**
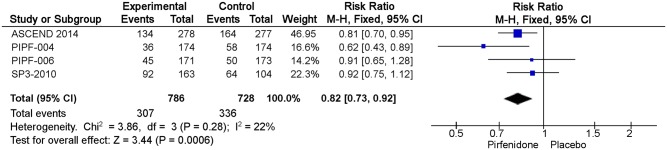
Comparison 3. Risk of progression (RR of PFS).
