# Supplementary material for: Pirfenidone for Idiopathic Pulmonary Fibrosis: A Systematic Review and Meta-Analysis
Source: PLoS One. 2015 Aug 26;10(8):e0136160. doi: 10.1371/journal.pone.0136160 (PMC4550327; doi:10.1371/journal.pone.0136160)
Supplement: S1 Table — (DOCX) [file pone.0136160.s012.docx]

S.1. Table. Excluded studies in this systematic review

| **Author** | **Year** | **Exclusion** |
| --- | --- | --- |
| Azuma et al. | 2011 | Sub-group analysis study |
| Raghu et al. | 1999 | Phase II open label study |
| Taniguchi et al. | 2011 | Sub-group analyses study |
